# Bacterial and protozoal pathogens found in ticks collected from humans in Corum province of Turkey

**DOI:** 10.1371/journal.pntd.0006395

**Published:** 2018-04-12

**Authors:** Djursun Karasartova, Ayse Semra Gureser, Tuncay Gokce, Bekir Celebi, Derya Yapar, Adem Keskin, Selim Celik, Yasemin Ece, Ali Kemal Erenler, Selma Usluca, Kosta Y. Mumcuoglu, Aysegul Taylan-Ozkan

**Affiliations:** 1 Department of Medical Microbiology, Hitit University, Corum, Turkey; 2 Department of Biology, Faculty of Arts and Science, Hitit University, Corum, Turkey; 3 National High Risk Pathogens Reference Laboratory, Public Health Institution of Turkey, Ankara, Turkey; 4 Department of Infectious Diseases and Clinical Microbiology, Hitit University, Corum, Turkey; 5 Department of Biology, Faculty of Science and Arts, Gaziosmanpasa University, Tokat, Turkey; 6 Emergency Medicine, Hitit University Corum Training and Research Hospital, Corum, Turkey; 7 Department of Emergency Medicine, Faculty of Medicine; Hitit University, Corum, Turkey; 8 National Parasitology Reference Laboratory, Public Health Institution of Turkey, Ankara, Turkey; 9 Parasitology Unit, Department of Microbiology and Molecular Genetics, The Kuvin Center for the Study of Infectious and Tropical Diseases, The Hebrew University-Hadassah Medical School, Jerusalem, Israel; 10 Department of Medical and Clinical Microbiology, Faculty of Medicine, Near East University, Nicosia, Northern Cyprus; Mahidol University, THAILAND

## Abstract

**Background:**

Tick-borne diseases are increasing all over the word, including Turkey. The aim of this study was to determine the bacterial and protozoan vector-borne pathogens in ticks infesting humans in the Corum province of Turkey.

**Methodology/Principal findings:**

From March to November 2014 a total of 322 ticks were collected from patients who attended the local hospitals with tick bites. Ticks were screened by real time-PCR and PCR, and obtained amplicons were sequenced. The dedected tick was belonging to the genus *Hyalomma*, *Haemaphysalis*, *Rhipicephalus*, *Dermacentor* and *Ixodes*. A total of 17 microorganism species were identified in ticks. The most prevalent *Rickettsia* spp. were: *R*. *aeschlimannii* (19.5%), *R*. *slovaca* (4.5%), *R*. *raoultii* (2.2%), *R*. *hoogstraalii* (1.9%), *R*. *sibirica* subsp. *mongolitimonae* (1.2%), *R*. *monacensis* (0.31%), and *Rickettsia* spp. (1.2%). In addition, the following pathogens were identified: *Borrelia afzelii* (0.31%), *Anaplasma* spp. (0.31%), *Ehrlichia* spp. (0.93%), *Babesia microti* (0.93%), *Babesia ovis* (0.31%), *Babesia occultans* (3.4%), *Theileria* spp. (1.6%), *Hepatozoon felis* (0.31%), *Hepatozoon canis* (0.31%), and *Hemolivia mauritanica* (2.1%). All samples were negative for *Francisella tularensis*, *Coxiella burnetii*, *Bartonella* spp., *Toxoplasma gondii* and *Leishmania* spp.

**Conclusions/Significance:**

Ticks in Corum carry a large variety of human and zoonotic pathogens that were detected not only in known vectors, but showed a wider vector diversity. There is an increase in the prevalence of ticks infected with the spotted fever group and lymphangitis-associated rickettsiosis, while *Ehrlichia* spp. and *Anaplasma* spp. were reported for the first time from this region. *B*. *microti* was detected for the first time in *Hyalomma marginatum* infesting humans. The detection of *B*. *occultans*, *B*. *ovis*, *Hepatozoon* spp., *Theileria* spp. and *Hemolivia mauritanica* indicate the importance of these ticks as vectors of pathogens of veterinary importance, therefore patients with a tick infestation should be followed for a variety of pathogens with medical importance.

## Author summary

Ticks are important vectors for different kind of pathogens, both of medical and veterinary importance, while tick-borne diseases (TBDs) are increasing all over the world. In Turkey, many important human and zoonotic TBDs such as, Lyme borreliosis, rickettsiosis, anaplasmosis, ehrlichiosis, tularemia, bartonellosis, babesiosis, theileriosis, and hepatozoonosis have been reported. Nonetheless, there is lack of research-based information concerning the epidemiology, ecology, and vector diversity of these tick-borne pathogens. In this study, we aimed to investigate broad-range bacterial and protozoan vector-borne pathogens by PCR/RT-PCR and sequencing, those ticks infesting humans in the Corum province. Spotted fever group rickettsiae and lymphangitis-associated rickettsiae, *Borrelia afzelii*, *Anaplasma* spp., *Ehrlichia* spp. were detected. *Babesia microti* was detected in *Hyalomma marginatum* infesting humans. Interestingly zoonotic pathogens like *Babesia ovis*, *Babesia occultans*, *Theileria* spp, *Hepatozoon felis*, *Hepatozoon canis*, and *Hemolivia mauritanica* were also detected, showing the role of ticks for diseases also of veterinary importance. This study provides important data for understanding the epidemiology of tick-borne pathogens and it is hoped that these results will challenge clinicians and veterinarians to unify their efforts in the management of TBDs.

## Introduction

Ticks are important vectors of a variety of diseases all over the world, including Turkey. They may transmit different kind of pathogens including bacteria, viruses, and protozoa affecting humans, domestic and wild animals [1,2]. Turkey is composed from a mosaic of habitats for ticks due to its diverse climate, vegetation, and large variety of wild and domestic animals [1,3]. Today, 48 tick species are known from this country, 31 of which have been found infesting humans [3].

Nineteen tick-borne diseases (TBDs) have been detected either in animals or humans in Turkey [1]. From 2002 to 2015, a total of 9,787 human cases of Crimean Congo hemorrhagic fever (CCHF) have been reported, 469 of which resulted in death [4]. Lyme borreliosis were reported in Turkey [5], while the sero-prevalence of *Borrelia burgdorferi* in humans was 4% [6]. Between 2005 and 2011, 4,824 human cases with tularemia were reported to the Ministry of Health [7]. Anaplasmosis is known from farm animals [8], while in humans, sero-positivity was 10.62% [9]. Ehrlichiosis and hepatozoonosis have been diagnosed in dogs [10,11]. The sero-prevalence for bartonellosis was 18.6% in cats [12], 6% in human blood donors [13], and 22.2% in cattle breeders and veterinarians [14]. Rickettsiosis was reported in Thrace and East Mediterranean regions of Turkey [15,16], the most prevalent being the Mediterranean Spotted Fever (MSF) [17]. Q fever cases in humans were reported from the Black Sea region of Turkey [18].

Babesiosis in animals is highly prevalent in Turkey, but there are no reports about clinical cases in humans [1]. Toxoplasmosis is one of the more common parasitic zoonosis worldwide, and in Turkey the prevalence in humans was found to vary between 13.9% and 76.6% [19]. Between the years 1988–2010, 50,381 cases of cutaneous leishmaniasis were reported to the Turkish Ministry of Health [20]. According to recent studies, ticks can be also possible vectors of toxoplasmosis and leishmaniasis [21,22].

The first CCHF cases in Turkey were observed in the province of Tokat which is a neighboring province of Corum; both cities are located in Kelkit Valley where the main vector, *Hyalomma marginatum* is prevalent [1,4]. Recently, 327 cases of CCHF and other TBDs such as rickettsial infections were reported from Corum [3,23–27]. The present study aims to investigate the human infested ticks species distribution; to determine their broad-ranges pathogens like *Rickettsia* spp., *Anaplasma* spp., *Ehrlichia spp*., *Coxiella burnetii*, *Borrelia burgdorferi* sensu lato, *Francisella tularensis*, *Bartonella* spp., *Leishmania* spp., *Toxoplasma gondii*, *Babesia* spp., *Theileria* spp., *Hepatozoon* spp., and *Hemolivia mauritanica* in Corum province of Turkey.

## Methods

### Study area

**Fig 1:**
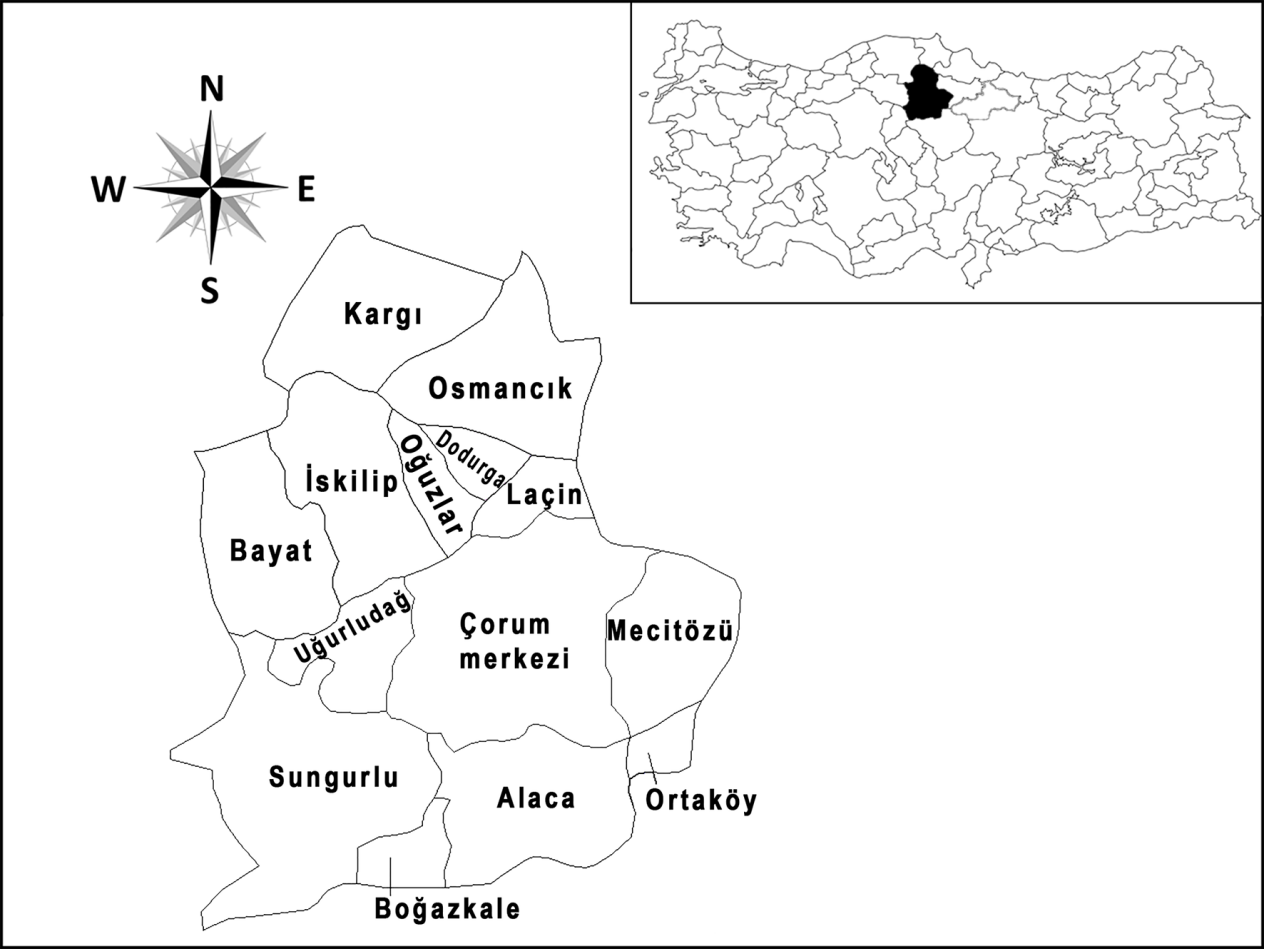
Map of the Corum province and its location within Turkey.

This study was carried out in the province of Corum (40° 33′ 00′′ N, 34° 57′ 14′′ E), which is located in Central Anatolia region of Turkey (Fig 1). It has a surface area of 12,820 km^2^, a population of 527,220 people, 152,244 of which live in the country site and another 374,926 in urban centers. The mean altitude is 801 m, the mean annual precipitation 429 mm, and the mean temperature 10–11°C. Due to the influences of the Black Sea and continental climates, the summers are hot and dry, while the winters are cold and rainy. Wild animals such as deer, boar, bear, badger, fox, rabbit, wolf, marten, squirrel and beaver are abundant throughout the province (Special Provincial Administration, Anonymous, 2009), while in rural areas farm animals are bred.

### Ticks collection and morphological identification

From March to November 2014 specimens were collected from patients who applied to the Emergency Service of the Hitit University Research and Training Hospital with a tick infestation. Ticks were morphologically identified under the stereomicroscope (Leica MZ16, Germany) using standard taxonomic keys [28–30].

### Amplification of tick-borne pathogen DNA

Individual ticks were mechanically homogenized by crushing with liquid nitrogen using disposable micro pestle and the DNA was extracted using the Tissue and Bacterial DNA Purification Kit (EURx DNA, Gdansk, Poland) according to the manufacturer’s protocols. All Polymerase Chain Reaction (PCR) amplifications were conducted with final volumes of 25 μl with 2.5 μl of template DNA, while negative and positive controls for each pathogen were used. With the exception of *Francisella tularensis* and protozoa, ticks were molecularly screened for pathogens by real-time-PCR using Evagreen master mix (Biotium, State, USA), while suspected samples were subjected to PCR. For the detection of *F*. *tularensis* and *Leishmania* a real-time-PCR taqman probe was used. For the identification of *Babesia*, the conventional PCR was used. All positive samples were sequenced. The primers BJ1 and BN2 amplifying *Babesia* spp., detected also *Theileria* spp., *Hepatozoon* spp. and *H*. *mauritanica*. The PCR methods, target genes and primer sequences used can be seen in Table 1 [31–41].

**Table 1:** PCR methods, target genes and primer sequences used for tick-borne pathogens.

Pathogen	Methods	Target gene	Primer sequences	Product size (bp)	Ref.
*Rickettsia* spp.	Real-time-PCR	23S rRNA,	PanR8F- AGC TTG CTT TTG GAT CAT TTG G PanR8R- TTC CTT GCC TTT TCA TAC ATC TAG T		31
*Rickettsia* spp.	PCR	ompA	Rr190.70p ATGGCGAATATTTCTCCAAAA Rr190.602n AGTGCAGCATTCGCTCCCCCT	532	32
*Rickettsia* spp.	PCR	gltA	RpCS.877p GGGGGCCTGCTCACGGCGG RpCS.1258n ATTGCAAAAAGTACAGTGAACA	381	32
*Anaplasma* spp., *Ehrlichia* spp.	realtime-PCR/, PCR	groEL	ESpF- TACTCAGAGTGCTTCTCAATGT ESpR- GCATACCATCAGTTTTTTCAAC	362	33
*Coxiella burnetii*	Real-time-PCR	ompA	CoxF- CAGAGCCGGGAGTCAAGCT CoxR- CTGAGTAGGAGATTTGAATCGC	82	34
*Bartonella* spp.	Real-time-PCR	ssrA	ssrA F-GCTATGGTAATAAATGGACAATGAAATAA ssrA R-GCTTCTGTTGCCAGGTG	301	35
*Borrelia* spp.	Real-time-PCR	16S rRNA	p16Swt F-GGATATAGTTAGAGATAATTATTCCCCGTTTG p16Swt R-CATTACATGCTGGTAACAGATAACAAGG	139	36
*Borrelia* spp.	PCR	flagellin	FL16- TGCTGGTGAGGGAGCTCAAGCTGCTCAGGCTGCACC TGTTCAAGAGGGTGCT FL17-TGCAGGTGAAGGCGCTCAGGCTGCTCCAGTGCAAGAGATAGGA	260	37
*Francisella tularensis*	Real-time-PCR taqman prob	tul4	Tul4F-ATTACAATGGCAGGCTCCAGA Tul4R- TGCCCAAGTTTTATCGTTCTTCT Tul4P- TTCTAAGTGCCATGATACAAGCTTCCCAATTACTAAG BHQ1)	91	38
*Babesia* spp. *Hepatozoon* spp, *Theileria* spp., *Hemolivia mauritanica*	PCR	18S rRNA	BJ1- GTCTTGTAATTGGAATGATGG BN2- TAGTTTATGGTTAGGACTACG	411–452	39
*Leishmania* spp.	Real-time -PCR taqman prob	ITS1 (ITS1 region between the SSU and 5.8S rRNA genes)	LITSR- CTGGATCATTTTCCGATG ITS1R- GAAGCCAAGTCATCCATCGC Probe: LC640-GCGGGGTGGGTGCGTGTGTG—PH	270–292	40
*Toxoplasma gondii*	Real-time-PCR /Evagreen	B1 gene	TOXO-F- TCCCCTCTGCTGGCGAAAAGT TOXO-R- AGCGTTCGTGGTCAACTATCGATTG	98	41

### Sequencing and phylogenetic analysis

PCR positive samples were purified and sequenced in one direction at a commercial sequencing service provider (Macrogen, Netherlands). Nucleotide sequences were analyzed using nucleotide Blast (National Centre for Biotechnology Information, www.blast.ncbi.nlm.nih.gov/Blast). Representative nucleotide sequences from this study were submitted to GenBank under accession numbers MF383491-MF383615 and MF494656-MF494660. A phylogenetic tree was constructed using the MEGA5.1 program.

## Results

A total of 322 ticks were collected from humans and identified as *Hyalomma marginatum* (n = 164, 50.9%), *Hyalomma excavatum* (n = 5; 1.5%), *Hyalomma aegyptium* (n = 1; 0.31%), *Hyalomma* spp. (n = 46; 14.3%), *Haemaphysalis parva* (n = 41; 12.7%), *Haemaphysalis punctata* (n = 6; 1.8%), *Haemaphysalis sulcata* (n = 1; 0.31%), *Rhipicephalus turanicus* (n = 34; 10.5%), *Rhipicephalus bursa* (n = 3; 0.93%), *Dermacentor marginatus* (n = 17; 5.2%) and *Ixodes ricinus* (n = 4; 1.24%). Overall, 37.2% of the examined ticks were infected with at least one pathogen; 3.7% of which with two different pathogens. The infection rate was 100% in *Dermacentor* spp., 89% in *Haemaphysalis* spp., 75% in *Ixodes* spp., 37% in *Hyalomma* spp. and 27% in *Rhipicephalus* spp. A total of 17 microorganism species were identified (Table 2). The most prevalent *Rickettsia* spp. being *R*. *aeschlimannii* (19.5%), *R*. *slovaca* (4.5%), *R*. *raoultii* (2.2%), *R*. *hoogstraalii* (1.9%), *R*. *sibirica* subsp. *mongolitimonae* (1.2%), *R*. *monacensis* (0.31%), and *Rickettsia* spp. (1.2%). In addition, the following pathogens were identified: *Borrelia afzelii* (0.31%), *Anaplasma* spp. (0.31%), *Ehrlichia* spp. (0.93%), *Babesia microti* (0.93%), *Babesia ovis* (0.31%), *Babesia occultans* (3.4%), *Theileria* spp. (1.6%), *Hepatozoon felis* (0.31%), *Hepatozoon canis* (0.31%), and *Hemolivia mauritanica* (2.1%). Table 3 shows the presence of bacterial pathogens according to the tick species, while in Table 4 the distribution of protozoan pathogens can be seen. All samples were negative for *Francisella tularensis*, *Coxiella burnetii*, *Bartonella* spp., *Toxoplasma gondii* and *Leishmania* spp.

**Table 2:** Total number and percentage of pathogens found in the 322 examined ticks, the percentage of their nucleotide identity and their accession number in NCBI GenBank.

Detected pathogens n / %		n / %	Nucleotide identity (%)	GenBank accession no.
*Rickettsia* spp. 100/31	*R*. *aeschlimannii*	63/19.5	99–100	MF383515- MF383577
	*R*. *slovaca*	15/4.6	99–100	MF383578- MF383592
	*R*. *raoultii*	7/2.2	99–100	MF383593- MF383599
	*R*. *hoogstraalii*	6/1.9	99–100	MF383600- MF383605
	*R*. *sibirica* subsp. *mongolitimonae*	4/1.2	99–100	MF383606- MF383609
	*R*. *monacensis*	1/0.31	98	MF383610
	*Rickettsia* spp.	4/1.2	90–99	-
*Ehrlichia* spp.		3/0.93	99–100	MF383611- MF383613
*Anaplasma* spp.		1/0.31	81	MF383615
*Borrelia afzelii*		1/0.31	100	MF383614
*Babesia* spp. 15/4.7	*B*. *microti*	3/0.93	99–100	MF383491- MF383493
	*B*. *occultans*	11/3.4	99–100	MF383494-MF383504
	*B*. *ovis*	1/0.31	99	MF383505
*Hepatozoon* spp. 2/0.62	*Hepatozoon canis*	1/0.31	99	MF383514
	*Hepatozoon felis*	1/0.31	99	MF383513
*Hemolivia mauritanica*		7/2.1	99–100	MF383506- MF383512
*Theileria* spp.		5/1.6	90–92	MF494656- MF494660

**Table 3:** Presence of bacterial pathogens in tick species isolated from humans in the Corum province.

Tick species	N	*R*. *aeschlimannii*	*R*. *slovaca*	*R*. *raoultii*	*R*. *hoogstraalii*	*R*. *sibirica* *subsp*. *mongolitimonae*	*R*. *monacensis*	*Rickettsia* spp.	*Ehrlichia* spp.	*Anaplasma* spp.	*B*. *afzelii*
*H*. *marginatum*	**164**	29	1	4	-	1	-	3	1		
*Hyalomma* spp.	**46**	11	1	1	-	-	-		1		
*H*. *excavatum*	**5**	-	-	-	-	1	-	-			
*H*. *aegyptium*	**1**	1									
*R*. *turanicus*	**34**	7	-	-	-	-	-	-			
*R*. *bursa*	**3**					1					
*Hae*. *parva*	**41**	9	2	-	4	1	-	1	1	1	
*Hae*. *punctata*	**6**	1			2						
*Hae*. *sulcata*	**1**	1	-	-	-	-	-	-			
*D*. *marginatus*	**17**	3	11	2							
*I*. *ricinus*	**4**	1	-	-	-	-	1	-			1
Total	**322**	**63**	**15**	**7**	**6**	**4**	**1**	**4**	**3**	**1**	**1**

**Table 4:** Presence of protozoan pathogens in tick species isolated from humans in the Corum province.

Tick species	N	*Babesia microti*	*Babesia occultans*	*Babesia ovis*	*Theileria* spp.	*Hepatozoon canis*	*Hepatozoon felis*	*H*. *mauritanica*
*H*. *marginatum*	164	3	10	-	2	-	-	-
*Hyalomma* spp. (nymph)	46	-	1	-	3	-	-	7
*R*. *turanicus*	34	-	-	-	-	-	1	-
*R*. *bursa*	3	-	-	1	-	-	-	-
*D*. *marginatus*	17	-	-	-	-	1	-	-
Total	322	3	11	1	5	1	1	7

## Discussion

**Fig 2:**
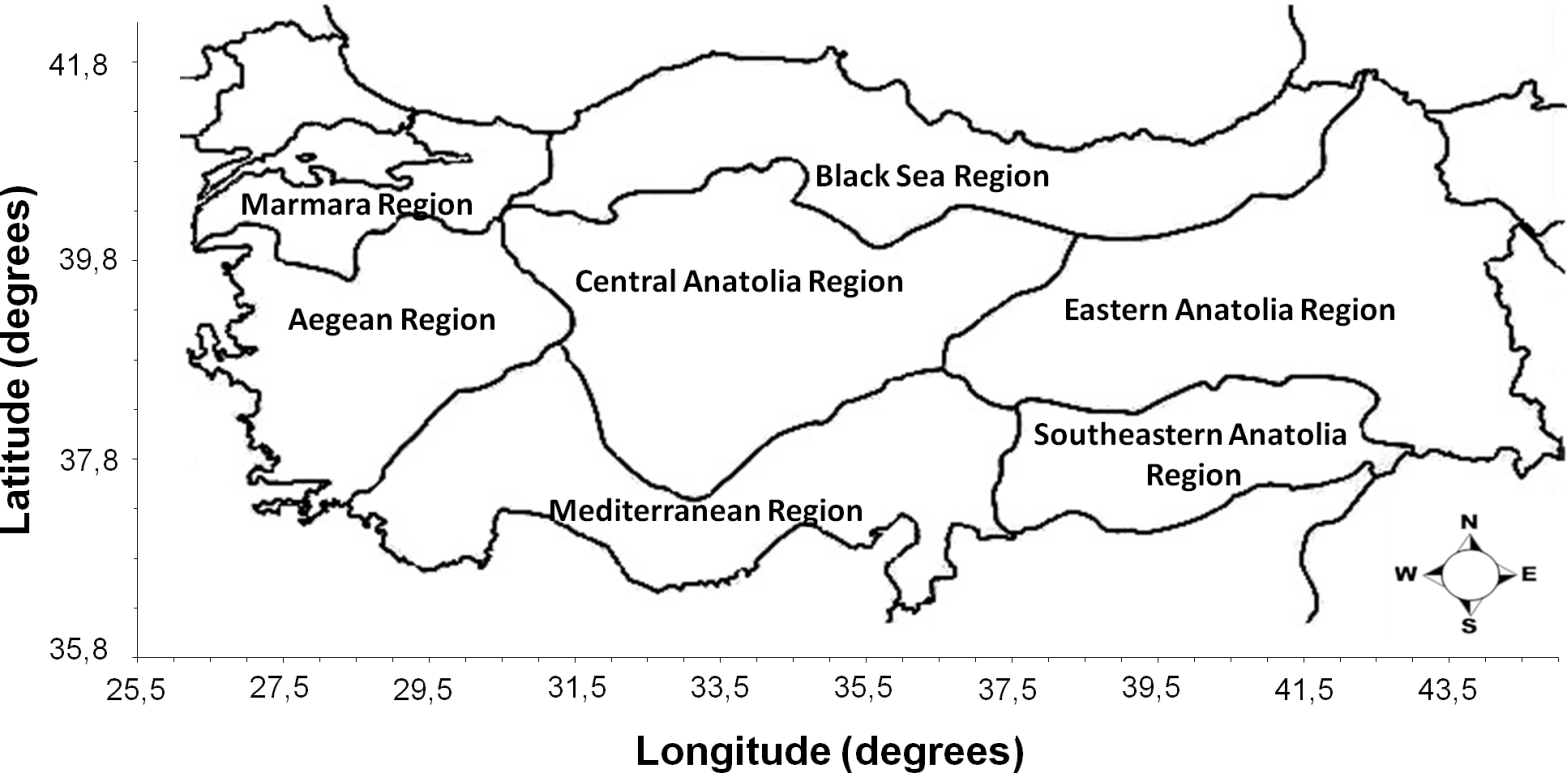
Seven main regions of Turkey.

Recently, a lot of attention is being given to ticks and tick-borne diseases in Turkey, were many individuals died as a result of CCHF [1,3,4]. Table 5 summarizes the studies done on ticks and their pathogens in the seven main regions of Turkey (Fig 2) [8,12,14,24–27,42–83].

**Table 5:** Tick-borne pathogens recorded in Turkey by regions.

**Marmara Region**				
**Provinces**	**Tick-borne pathogens**	**Method**	**Hosts**	**Ref.**
Istanbul	*R*. *monacensis*, *R*. *aeschlimannii*, *R*. *conorii* subsp. *conorii*, *R*. *helvetica*, *R*. *raoultii*, *R*. *africae*, *R*. *felis*	Nested PCR	Ticks (*I*. *ricinus*, *R*. *sanguineus*, *H*. *aegyptium*, *Hyalomma* spp., *H*. *marginatum*, *D*. *marginatus*)	24
	*Rickettsia* spp, *B*. *burgdorferi* s.l.	Semi Nested PCR	Ticks (*D*. *marginatus*, *H*. *aegyptium*, *H*. *aegyptium*, *Haemaphysalis* spp., *Ixodes* spp., *I*. *ricinus*, *R*. *bursa*, *R*. *sanguineus* gr.)	42
Thrace region	*R*. *conorii*	PCR in skin biopsies	Human	43
Thrace (including a recreational park Zekeriyakoy, Belgrad Forest in the Istanbul metropolitan area)	*B*. *burgdorferi* s.s., *B*. *garinii (Eurasian type)*, *B*. *afzelii*, *B*. *lusitaniae*, *B*. *valaisiana*	PCR	Ticks (*I*. *ricinus*)	44
Istanbul	*B*. *canis*, *B*. *vogeli*, *B*. *rossi*	PCR	Dogs	25
Adana, Aydin, Bursa, Hatay, Istanbul Urfa Kars, Kirikkale Sivas,	*B*. *vinsonii* subsp. *berkhoffii*	IFA	Dogs	45
Istanbul	*F*. *tularensis*	Microagglutination	Human	46
Istanbul, Kirklareli	*A*. *phagocytophilum*, *B*. *burgdorferi* s.l.	PCR	Ticks (*I*. *ricinus*)	47
**Agean Region**				
**Provinces**	**Tick-borne pathogens**	**Method**	**Hosts**	**Ref**
Aydin	*T*. *annulata*	IFA	Cattle	48
Aydin and Denizli	*B*. *henselae*	IFA	Human	14
Aydin	*A*. *centrale*, *A*. *marginale*, *A*. *phagocytophilum*	PCR	Cattle, Ticks (*H*. *marginatum*, *H*. *excavatum*)	49
Adana, Aydin, Bursa, Hatay, Istanbul, Urfa Kars, Kirikkale, Sivas	*B*. *vinsonii* subsp. *berkhoffii*	IFA	Dogs	45
Manisa	West Nile virus, CCHFV, *F*. *tularensis*, *B*. *burgdorferi*	ELISA, IFA, WB	Human	50
**Central Anatolia Region**				
**Provinces**	**Tick-borne pathogens**	**Method**	**Hosts**	**Ref**
Kirklareli	*A*. *marginale*	Nested PCR	Cows	51
Ankara	*B*. *crassa*, *B*. *major*, *B*. *occultans*, *B*. *rossi*, *B*. *burgdorferi* s.s., *R*. *aeschlimannii*, *R*. *slovaca*, *R*. *hoogstraalii*	PCR and sequencing analysis.	Ticks (*Haemaphysalis*, *Hyalomma*, *Ixodes Rhipicephalus*)	26
	*B*. *caballi*, *B (T*.*)*. *equi*	PCR	Horses	52
	*E*. *canis*		Dogs	53
	*B*. *vinsonii* subsp. *berkhoffii*			54
Kayseri	*E*. *canis*, *B*. *canis canis*, *B*. *gibsoni*, *A*. *phagocytophilum*, *H*. *canis*, *B*. *canis vogeli*	Real Time PCR	Dogs	55
Yozgat	*R*. *aeschlimannii*, *R*. *hoogstraalii*, *R*. *raoultii*, *R*. *slovaca*	PCR	Ticks (*H*. *marginatum*, *H*. *parva*, *D*. *marginatus*)	56
	*F*. *tularensis*	Microagglutination	Human	57
Ankara	*B*. *henselae*, *Bartonella clarridgeiae*	Culture	Cats	12
Ankara	*B*. *ovis*, *T*. *ovis*, CCHFV	PCR	Anatolian wild sheep and ticks (*Rh*. *bursa*, *H*. *excavatum*)	58
Konya	*B*. *canis vogeli*, *H*. *canis*, *Hepatozoon* sp. MF, *Mycoplasma*. *haemocanis*, *M*. *haematoparvum*	PCR	Dogs	59
Konya	*B*. *ovis*	IFAT	Sheep	60
Sivas	*B*. *bigemina*, *B*. *bovis*	IFAT	Cattle	61
Sivas, Amasya	*Rickettsia* spp., *Francisella*, *Coxiella*, *Neisseriaceae*, *Enterobacteriaceae*, *Francisella*, *Coxiella*, *Shigella*	PCR	Ticks (*R*. *(B*.*) annulatus*, *D*. *marginatus*)	62
Adana, Aydin, Bursa, Hatay, Istanbul Urfa Kars, Kirikkale Sivas,	*B*. *vinsonii* subsp. *berkhoffii*	IFA	Dogs	45
**Black Sea Region**				
**Provinces**	**Tick-borne pathogens**	**Method**	**Hosts**	**Ref**
Bolu, Kastamonu, Corum, Samsun, Tokat, Giresun, Bayburt provinces of the Black Sea region of Turkey	*T*. *ovis*, *B*. *ovis*, *B*. *bigemina*, *B*. *microti*	PCR	Ticks (*R*. *bursa*, *R*. *turanicus*, *R*. *sanguineus*, *H*. *parva*, *H*. *marginatum*, *I*. *ricinus*)	63
Sinop	*B*. *microti*	IFA	Human	64
Middle and Eastern Black Sea	*A*. *phagocytophilum*	IFAT, PCR, microscopy	Sheep and cattle	8
Tokat, Amasya, Gumushane, Giresun, Trabzon, Rize.	*T*. *annulata*, *T*. *buffeli/orientalis* *B*. *bigemina*, *B*. *major*, *Babesia* sp.	reverse line blot	Cattle	65
Bartin	*B*. *bovis*, *B*. *bigemina*, *B*. *divergens*, *B*. *occultans*	reverse line blot	Cattle and ticks (*R*. *(B*.*) annulatus*)	66
Giresun, Trabzon, Rize	*A*. *phagocytophilum*	Nested PCR	Ticks (*I*. *ricinus*, *Ixodes* spp.)	67
Giresun, Trabzon, Rize, Tokat, Amasya, Gumushane	*A*. *marginale*, *A*. *centrale*, *A*. *phagocytophilum*, *A*. *ovis*, *Ehrlichia*	PCR	Cattle	68
Giresun, Trabzon, Rize, Tokat, Amasya, and Gumushane	*T*. *buffeli/orientalis*, *Babesia* spp., *Anaplasma/Ehrlichia* spp., *A*. *centrale*, *A*. *phagocytophilum*	PCR	Ticks (*R*. *bursa*, *R*. *(B*.*) annulatus*, *H*. *excavatum*, *H*. *marginatum*)	69
Ordu	*C*. *burnetii*	IFAT IgG	Human	70
Sivas, Amasya,	*Rickettsia* spp., *Francisella*, *Coxiella*, *Neisseriaceae*, *Enterobacteriaceae*, *Shigella*	PCR	Ticks (*R*. *(B*.*) annulatus*, *D*. *marginatus*)	62
Corum	*R*. *aeschlimannii*, *R*. *sibirica mongolitimonae*, *R*. *raoultii*, *R*. *slovaca*	PCR	Ticks (*H*. *marginatum*, *D*. *marginatus*)	27
Tokat	*R*. *aeschlimannii*, *R*. *sibirica mongolitimonae*	PCR	Ticks (*H*. *marginatum*)	71
**Eastern Anatolia Region**				
**Provinces**	**Tick-borne pathogens**	**Method**	**Hosts**	**Ref**
Erzincan	*T*. *annulata*, *T*. *buffeli/orientalis*	reverse line blotting	Cattle	72
Kars	*B (T)*. *equi*	IFA	Horses	73
Igdir	*E*. *canis*	ELISA	Dogs	74
Elazig, Malatya, Mus Tunceli, Bingol, Bitlis,	*C*. *burnetii*	PCR	Sheep	75
Elazig	*Ehrlichia* spp., *A*. *platys*, *A*. *ovis*	PCR & sequence	Ticks (*H*. *anatolicum*, *R*. *bursa*, *R*. *sanguineus*)	76
Erzincan	*C*. *burnetii*	ELISA	Human	77
Erzurum	*B*. *canis*, *Hepatozoon* spp., *H*. *canis*, *D*. *immitis*, *E*. *canis*	Nested PCR	Dogs	78
Elazig	*B*. *ovis*	PCR	Sheep, goats, ticks (*R*. *bursa*)	79
Erzurum	*T*. *equi*, *B*. *cabali*	Multiplex PCR	Horses	80
**Souteastern Anatolia Region**				
**Provinces**	**Tick-borne pathogens**	**Method**	**Hosts**	**Ref**
Adana Gaziantep Adiyaman	*Babesia ovis*, *Theileria annulata*	PCR	Ticks (*R*. *bursa*, *R*. *turanicus*, *H*. *excavatum*, *H*. *parva*, *H*. *anatolicum*)	81
Diyarbakir	*Babesia* sp., *B*. *canis*, *B*. *vogeli*, *H*. *canis*	Nested PCR	Dogs	82
	*H*. *canis*, *H*. *felis *	PCR	Ticks (*R*. *sanguineus*)	83
Adana, Aydin, Bursa, Hatay, Istanbul Urfa Kars, Kirikkale Sivas,	*B*. *vinsonii* subsp. *berkhoffii*	IFA	Dogs	45

In Corum province, 10 tick species infesting humans were identified, the most prevalent being *H*. *marginatum*, *Hae*. *parva*, *R*. *turanicus* and *D*. *marginatus*. Similar results from the same region has been obtained by Keskin et al., [84, 85], who, in addition to the tick species found in the present study, also reported the infestation of humans with *Haemaphysalis erinacei taurica* and *Ixodes laguri*. In their study the most prevalent tick species isolated from humans were *H*. *marginatum*, *D*. *marginatus*, *R*. *turanicus* and *R*. *bursa*. The differences could be explained with the changes in tick abundance according to climatic conditions, host factors, socio-demographic factors, deforestation, as well as agricultural and wildlife management [86].

In the present study all *D*. *marginatus* specimens were infected with at least one pathogen, while the infection rate was high also in *Haemaphysalis* spp. Orkun et al. who investigated tick pathogens in Ankara province found high infection rate of *Rickettsia* spp., *Babesia* spp., and *Borrelia* spp. in the same tick species [26].

*Rickettsia* spp. was identified as the most prevalent tick-borne pathogen in this study (31%). Other studies reported an average infection rate of 41.3 in Istanbul [24], while in Yozgat province the rate was 10.5% [56], and in Ankara province 27.2%[26].

*Rickettsia aeschlimannii* is commonly transmitted by *Hyalomma* and *Rhipicephalus* spp. [2]. In Turkey, *R*. *aeschlimannii* was detected in *H*. *marginatum*, *H*. *aegyptium*, *H*. *excavatum*, *R*. *bursa* and *R*. *turanicus* ticks [24,26,56,87,88]. In our study, this pathogen was found in all tick species examined with the exception of *H*. *excavatum* and *R*. *bursa*. To the best of our knowledge, this is the first report that *R*. *aeschlimannii* was found in *Haemaphysalis* spp., *Dermacentor* spp., and *Ixodes* spp. ticks, indicating that the pathogen might be transmitted also by other tick species. According to nucleotide Blast and phylogenetic analysis (*ompA*) (Annex 1), *R*. *aeschlimannii* strains in our study is closely related with *R*. *aeschlimannii* isolate BB-35/Camli-H.marg (99–100% identity, accession number KF791251).

*Rickettsia aeschlimannii* was the most prevalent (19.5%) pathogen among *Rickettsia*-positive ticks in this study. In an investigation which was performed in 2009 in Corum province, *R*. *aeschlimannii* was found in 5% of the ticks [87], while in Ankara and Yozgat provinces, where similar climatic conditions prevail, this pathogen was detected in 4.7% and 4.3%, respectively of ticks examined [26,56]. It was reported that *R*. *aeschlimannii* infections exhibited symptoms similar to Mediterranean spotted fever (MSF) [89], and potentially lead to severe symptoms resembling to those of viral hemorrhagic fever [17]. Accordingly, *R*. *aeschlimannii* infection should be included in the differential diagnosis, especially in endemic regions of MSF.

*Rickettsia slovaca* is usually transmitted by *Dermacentor* ticks and is associated with symptoms characterized by inoculation eschar on the scalp, necrosis erythema and cervical lymphadenopathy [2,24,56,88,90]. This disease is either called tick-borne neck lymphadenopathy (TIBOLA) or *Dermacentor*-borne necrosis erythema and lymphadenopathy (DEBONEL) [90]. Incidence of *R*. *slovaca* infections is likely underestimated. Parola et al. reported that in 49 out of 86 (57%) TIBOLA/DEBONEL cases the etiologic agent was *R*. *slovaca* [90]. Throughout Europe, *Dermacentor marginatus* and *Dermacentor reticulatus* ticks are responsible from transmission of this pathogen [90]. In our study, in addition to *Dermacentor* spp. ticks, this pathogen was for the first time also detected in *H*. *marginatum*, *Hyalomma* spp. nymphs and *Hae*. *parva* (Table 3). Nucleotide Blast and phylogenetic analysis (*ompA*,) of *R*. *slovaca* Corum strains were 99% identical to *R*. *slovaca* isolate BB-51/Akyurt-D.marg (accession number KF791235) (Annex 1), while the *gltA* gene of *R*. *slovaca* Corum strains (Annex 2), showed a 99% identity to *R*. *slovaca* strain PotiR30 (accession number DQ821852). In the present study *R*. *slovaca* was detected in 4.6% of the ticks. In similar studies conducted earlier, *R*. *slovaca* was found in 0.3% of ticks in Corum [87], in 4.8% in Yozgat province [56], and in 9.4% in Ankara province [26].

Similar to *R*. *slovaca*, *R*. *raoultii* is also the etiological agent of TIBOLA/DEBONEL and this *Rickettsia* seems to be less pathogenic and less frequent than *R*. *slovaca* [90]. Parola et al reported that in 7 out of 86 (8%) TIBOLA/DEBONEL cases the etiologic agent was *R*. *raoultii* [90]. *Dermacentor* ticks are known vectors of *R*. *raoultii* [24,56,88]. In the present study, in addition to *Dermacentor* spp., *R*. *raoultii* was also found in *H*. *marginatum* and *Hyalomma* spp. nymphs (Table 3). The nucleotide Blast and phylogenetic analysis of *gltA* gene of Corum *R*. *raoultii* strains (Annex 2) share a 99% sequence identity to *R*. *raoultii* clone Ds1 (accession number KF003009) and accordingly to *ompA* genes (Annex 1). In addition, a 99% similarity was found to *R*. *raoultii* strain WB16/Dm Monterenzio (accession number HM161789). *Rickettsia raoultii* was detected in 2.2% of the ticks examined. Earlier studies from Corum reported that the percentage was 0.3% [27] and in Yozgat province 0.4% [56], while this rickettsia was not detected in ticks from the Ankara region [26]. In Corum province, the rate of *R*. *slovaca and R*. *raoultii* in ticks infesting humans increased in comparison to 2009, and it seems that these pathogens are extending their vector diversity.

*Rickettsia hoogstraalii* has an unknown pathogenicity and it is transmitted by *Hae*. *Parva* [26,56,88], however, we found it in *Hae*. *parva* and *Hae*. *punctata* ticks. The nucleotide Blast and phylogenetic analysis of *gltA* gene of Corum *R*. *hoogstraalii* strains (Annex 2) have a 99% similarity to *R*. *hoogstraalii* strain RCCE3 with accession number EF629539. In our study the prevalence of *R*. *hoogstraalii* was 1.9%, while in Yozgat was 0.87% [56], and in Ankara 13% [26].

*Rickettsia sibirica* subsp. *mongolitimonae*, symptoms are characterized by fever, eschar and lymphadenopathies [91] and it is transmitted by ticks such as *Hyalomma asiaticum*, *Hyalomma truncatum*, *H*. *excavatum* and *R*. *bursa* [2,91–93]. We found this pathogen in *H*. *marginatum*, *H*. *excavatum*, *R*. *bursa*, and *Hae*. *parva* ticks. To the best of our knowledge this is the first detection of this pathogen in *Hae*. *parva* ticks. Nucleotide Blast and phylogenetic analysis of *R*. *sibirica* subsp. *mongolitimonae* Corum strains (*ompA*) (Annex 1), showed a 99% identity to *R*. *sibirica* subsp. *mongolitimonae* Bpy1 (accession number KT345980). In this study this *Rickettsia* species was detected earlier in 1.2% of the ticks, while it was reported in 0.3% of *H*. *marginatum* ticks in Corum [87] and in 0.25% of ticks in Tokat province [71].

*Rickettsia monacensis* infection shows flu-like symptoms, eschar and rash, the main vector of this pathogen being *Ixodes ricinus* [91]. In Anatolian region of Turkey this tick species is rare [3]. The *ompA* genes of Corum *R*. *monacensis*, which was detected also in our study in *I*. *ricinus* ticks, showed 99% identity with *R*. *monacensis* isolate Est1623 (accession number KT119437) (Annex 1). In previous studies this pathogens was not found in the Ankara and Yozgat provinces [26,56], whereas the infection rate was 30.5% in ticks infesting humans in Istanbul [24]

*Ehrlichia* spp. effect both humans and animals such as dogs and domestic ruminants with symptoms like fever, malaise, leucopenia, thrombocytopenia, and abnormal liver function [94]. The vectors of this pathogen are *Amblyomma*, *Dermacentor*, *Rhipicephalus*, *Ixodes* and *Haemaphysalis* ticks [2,94]. In this study, *Ehrlichia* spp. were detected in 0.93% of *H*. *marginatum*, *Hyalomma* spp. nymphs and *Hae*. *parva*. Nucleotide Blast and phylogenetic analysis of *groEL* genes of Corum *Ehrlichia* spp. strain (Annex 3) was 99% identical to *Ehrlichia ewingii* isolate AaFT81 GroEL.

In Turkey, bovine anaplasmosis was detected in *I*. *ricinus* ticks which were collected from cattle in the cost of Black Sea [67]. In the present study, *Anaplasma* spp. was found in *Hae*. *parva* ticks. Nucleotide Blast and phylogenetic analysis of *groEL* genes of Corum *Anaplasma* spp. strain shared an 81% identity to *Anaplasma phagocytophilum* isolate Omsk-vole52 with accession number KF745743, (Annex 3).

*Coxiella burnetii* is the etiological agent of Q-fever with flu-like symptoms and is considered as a zoonotic disease. The role of ticks in the transmission of *C*. *burnetii* to humans is low [95]. In present study this pathogen was not detected in ticks infesting humans.

*Borrelia afzelii* is the pathogenic agent of Lyme disease transmitted mainly by ticks belonging to the genus *Ixodes*. This pathogen is known from Europe, western parts of the former USSR and Northern Africa [2]. We detected it in one *I*. *ricinus* specimen. According to *flagelline* gene sequence analyses *B*. *afzelii* Corum strain was 100% identical to *B*. *afzelii* strain S60 with accession number KM198345 (Annex 4). Orkun et al. reported the presence of *Borrelia burgdorferi* sensu stricto in 3.5% of *Hyalomma* spp. and *Hae*. *parva* in Ankara province [26]. Lyme disease pathogens are prevalent in Istanbul region which has a moderate and wet climate and the infection rate in ticks collected from different areas was 38.7% [47]. *Francisella tularensis* is the causative agent of tularemia a severe zoonotic diseases affecting animals and humans. This pathogen was isolated from the bird-rabbit tick, *Haemaphysalis leporispalustris* [95] and from *Dermacentor reticulatus* infesting red foxes [96]. In Turkey, tularemia cases were generally transmitted as water-borne but there are few tick-borne cases [46,57,97]. *F*. *tularensis* was neither found in ticks collected from several barns, cattle and people [98], nor in the ticks of the present study.

*Bartonella* spp. are zoonotic vector-borne infection agents of humans. One of them, *B*. *henselae* is the pathogenic agent of cat-scratch disease, the main vector being the cat flea (*Ctenocephalides felis*) [12], however a direct link between tick bites, *B*. *henselae* and disease symptoms was reported in humans [99]. In the present study *B*. *henselae* was not detected in any of the ticks examined.

*Babesia* spp. are the pathogenic agents of babesiosis in humans and animals, which are considered as emerging diseases worldwide [86]. In Europe, infection rates of *Babesia* spp. in ticks ranges from 0.9 to 20% [100]. *B*. *microti* is pathogenic to humans causing malaria-like symptoms. The geographical distribution of this pathogen is USA, Canada, and Europe while the main vector is *Ixodes* spp. [2,100]. In USA, the prevalence of *B*. *microti* in ticks was 8.4% [101], while in ticks collected from vegetation in Poland was 2.8% [102]. In addition to *Ixodes* spp., *B*. *microti* was also detected in 0.7% of *Dermacentor reticulatus* in Switzerland [39]. In Turkey, *B*. *microti* was for the first time detected in one *I*. *ricinus* tick collected from a ruminant [63]. In Sinop province of Turkey, the sero-prevalence of *B*. *microti* in humans was 6.23% [64], while in the present study, the prevalence of *B*. *microti* in *H*. *marginatum* ticks was 0.93%. According to *18SrRNA* gene nucleotide Blast and phylogenetic analysis, *B*. *microti* Corum strains were 100% identical to *B*. *microti* isolate RUS/Nov15-2950-Ipr with accession number KX987864 (Annex 5). This is the first report showing the presence of *B*. *microti* in *H*. *marginatum* infesting humans, which is the most prevalent tick species in Corum province and is the main vector for *B*. *microti*.

*Babesia occultans* is a bovine parasite with high prevalence in South Africa, the vectors being *Hyalomma* spp. [2]. In Turkey, presence of *B*. *occultans* was reported by Aktas et al. in *H*. *marginatum* and *R*. *turanicus* collected from the vegetation, agricultural fields and grazing cattle, with a prevalence rate of 7%; transstadial and transovarial transmission of *B*. *occultans* were also demonstrated [103]. Orkun *et al*. reported this pathogen in 0.6% of *H*. *marginatum* infesting humans [26]. In our study *B*. *occultans* was present in 3.4% of *H*. *marginatum*, strongly supporting the presence of this pathogen not only in ticks infesting animals but also humans. The *18SrRNA* genes of Corum *B*. *occultans* strains showed a 99% similarity to *B*. *occultans* isolate Trender1with accession number KP745626 (Annex 5).

*Babesia ovis* is the causative agent of sheep babesiosis and mainly prevalent in Africa, Asia, and Europe, the vectors of this pathogen are *R*. *bursa* and *R*. *turanicus* [2]. In Turkey, in ticks collected from sheep and goats the prevalence was 16.37% [79]. *B*. *ovis* was detected by us in one *R*. *bursa* infesting a patient. According to *18SrRNA* gene nucleotide Blast and phylogenetic analyses (Annex 5), *B*. *ovis* Corum strains was 99% identical to *B*. *ovis* isolate tick20.3D with accession number KT587794 (Annex 5).

Recent studies show that ticks collected from cats and dogs may be responsible for the transmission of *Toxoplasma gondii* [21]. *Leishmania infantum* was also found on ticks infesting dogs [22]. In our study, these agents could not be detected.

*Hepatozoon canis* and *Hepatozoon felis* are the causative agents of hepatozoonosis in dogs and cats. These pathogens are transmitted by *Rhipicephalus sanguineus*, *Hae*. *longicornis*, and *R*. *turanicus* [2]. In Turkey, *H*. *canis* and *H*. *felis* were for the first time identified in *R*. *sanguineus* ticks removed from dogs [83], while *H*. *canis* infection was also reported in dogs [104]. We demonstrated the presence of *H*. *canis* in *D*. *marginatus* and of *H*. *felis* in *R*. *turanicus*. The *18SrRNA* genes of Corum *H*. *canis* strain showed a 99% similarity to *H*. *canis* isolate 204B/13b (accession number KP216425), while the Corum *H*. *felis* strain showed a 99% similarity to *H*. *felis*, clone 8533, accession number KC138533 (Annex 5).

*Theileria* spp. are the pathological agents of theileriosis of ruminants, equids and felids, the vectors being ticks from the genera *Hyalomma* and *Rhipicephalus* [1,2]. A transstadial but not transovarial transmission was reported in these ticks [105]. In our study *Theileria* spp. was demonstrated in *Hyalomma* spp. infesting humans and the prevalence rate was 1.6%. According to *18SrRNA* genes, the Corum strain of *Theileria* spp showed a 92% similarity to *Theileria youngi* (accession number AF245279) (Annex 5).

*Hemolivia mauritanica* is a pathogen of tortoises and transmitted by *H*. *aegyptium* [106]. In the present study, this pathogen was found only in *Hyalomma* spp. nymphs infesting humans and the prevalence rate was 2.1%. According to *18SrRNA* genes, Corum *H*. *mauritanica* strains showed a 99% similarity to *H*. *mauritanica* isolate SY-45-10 (accession number KF992707 (Annex 5).

In conclusion, ticks in Corum province carry a large variety of human and zoonotic pathogens. There are indications showing that there is an increase in the rate of ticks carrying spotted fever group and lymphangitis-associated *Rickettsiae*, while *Ehrlichia* spp. and *Anaplasma* spp. were reported for the first time in the region. To the best of our knowledge *B*. *microti* was detected for the first time in *H*. *marginatum* infesting humans. The presence of pathogens such as *B*. *occultans*, *B*. *ovis*, *Hepatozoon* spp., *Theileria* spp. and *H*. *mauritanica* show the role of ticks for diseases of veterinary importance. Pathogens are detected not only in ticks known as vectors but in a variety of other ticks, indicating wider vector diversity. Patients with a tick bite history in Corum region should be followed not only for CCHF but also for other pathogens of medical importance.

## Supporting information

S1 Fig Phylogenetic tree of rickettsial *ompA* gene. Phylogenetic tree based on aligned sequences of the rickettsial ompA gene, constructed using UPMGA in MEGA5.1 software. GenBank accession numbers of the *Rickettsiae* are given after the names of bacteria.
(TIF)

S2 Fig Phylogenetic tree of rickettsial *gltA* gene. Phylogenetic tree based on aligned sequences of the rickettsial gltA gene, constructed using UPGMA in MEGA5.1 software. GenBank accession numbers of sequences are given after the names of bacteria.
(TIF)

S3 Fig Phylogenetic tree of *Ehrlichia* heat shock protein (*groEL*) gene. Phylogenetic tree based on aligned sequences of the heat shock protein (*groEL*) gene, constructed using UPGMA in MEGA5.1 software. GenBank accession numbers of sequences are given after the names of bacteria.
(TIF)

S4 Fig Phylogenetic tree of *Borrelia flaB* gene. Phylogenetic tree based on aligned sequences of the *Borrelia flaB* gene, constructed using UPGMA in MEGA5.1 software. GenBank accession numbers of sequences are given after the names of bacteria.
(TIF)

S5 Fig Phylogenetic tree of *18S ribosomal RNA* gene. Phylogenetic tree based on aligned sequences of *18S ribosomal RNA* gene, constructed using UPGMA in MEGA5.1 software. GenBank accession numbers of sequences are given after the names of the protozoa.
(TIF)

## References

[1] Inci A, Yildirim A, Duzlu O, Doganay M, Aksoy S. Tick-borne diseases in Turkey: A review based on one health perspective. PLoS Negl Trop Dis. 2016;10 (12):e0005021 doi: 10.1371/journal.pntd.0005021 27977689 10.1371/journal.pntd.0005021 PMC5158090

[2] de la Fuente J, Estrada-Pena A, Venzal JM, Kocan KM, Sonenshine DE. Overview: Ticks as vectors of pathogens that cause disease in humans and animals. Front Biosci. 2008;13:6938–46. 18508706 10.2741/3200

[3] Bursali A, Keskin A, Tekin S. A review of the ticks (Acari: Ixodida) of Turkey: Species diversity, hosts and geographical distribution. Exp Appl Acarol. 2012; 57(1):91–104. doi: 10.1007/s10493-012-9530-4 22371208 10.1007/s10493-012-9530-4

[4] Leblebicioglu H, Ozaras R, Irmak H, Sencan I. Crimean-Congo hemorrhagic fever in Turkey: Current status and future challenges. Antiviral Res. 2016;126:21–34. doi: 10.1016/j.antiviral.2015.12.003 26695860 10.1016/j.antiviral.2015.12.003

[5] Polat E, Turhan V, Aslan M, Musellim B, Onem Y, Ertugrul B. First report of three culture confirmed human Lyme cases in Turkey. Mikrobiyol Bul. 2010;44(1):133–9 (in Turkish). 20455410

[6] Aslan Basbulut E, Gozalan A, Sonmez C, Coplu N, Korhasan B, Esen B, et al Seroprevalence of *Borrelia burgdorferi* and tick-borne encephalitis virus in a rural area of Samsun, Turkey. Mikrobiyol Bul. 2012;46:247–56 (in Turkish). 22639313

[7] Doganay M. Tularaemia: Re-Emerging Disease. In: Proceedings of 1st National Symposium on Vectors and Vector Borne Diseases with International Participation, 9–10 September, 2012, Avanos, Cappadocia, Nevsehir, Turkey pp. 62–4.

[8] Gokce HI, Genc O, Akca A, Vatansever Z, Unver A, Erdogan HM. Molecular and serological evidence of *Anaplasma phagocytophilum* infection of farm animals in the Black Sea region of Turkey. Acta Vet Hung. 2008;56(3):281–92. doi: 10.1556/AVet.56.2008.3.2 18828480 10.1556/AVet.56.2008.3.2

[9] Gunes T, Poyraz O, Atas M, Turgut NH. The seroprevalence of *Anaplasma phagocytophilum* in humans from two different climatic regions of Turkey and its co-seroprevalence rate with *Borrelia burgdorferi*. Turk J Med Sci. 2011;41:903–8. doi: 10.3906/sag-1009-1148

[10] Ulutas B, Bayramli G, Karagenc T. First case of *Anaplasma* (*Ehrlichia*) *platys* infection in a dog in Turkey. Turk J Vet Animal Sci. 2007;31:279–82.

[11] Karagenc TI, Pasa S, Kirli G, Hosgor M, Bilgic HB, Ozon YH, et al A parasitological, molecular and serological survey of *Hepatozoon canis* infection in dogs around the Aegean coast of Turkey. Vet Parasitol. 2006;135:113–9. doi: 10.1016/j.vetpar.2005.08.007 16229952 10.1016/j.vetpar.2005.08.007

[12] Celebi B, Kilic S, Aydin N, Tarhan G, Carhan A, Babur C. Investigation of *Bartonella henselae* in cats in Ankara, Turkey. Zoonoses Public Health. 2007;56:169–75. doi: 10.1111/j.1863-2378.2008.01170.x 18990198 10.1111/j.1863-2378.2008.01170.x

[13] Yilmaz C, Ergin C, Kaleli I. Investigation of *Bartonella henselae* seroprevalence and related risk factors in blood donors admitted to Pamukkale University blood center. Mikrobiyol Bul. 2009;43:391–401 (in Turkish). 19795614

[14] Sayin-Kutlu S, Ergin C, Kutlu M, Akkaya Y, Akalin S. *Bartonella henselae* seroprevalence in cattle breeders and veterinarians in the rural areas of Aydin and Denizli, Turkey. Zoonoses Public Health. 2012;59:445–9. doi: 10.1111/j.1863-2378.2012.01486.x 22489645 10.1111/j.1863-2378.2012.01486.x

[15] Kuloglu F, Akata F, Tansel O, Gurcan S, Sakru N, Otkun M, et al Serologically confirmed cases of Mediterranean Spotted Fever in the Trakya region of Turkey. Turkiye Parazitol Derg. 2004;28 (3):167–70.

[16] Kuscu F, Orkun O, Ulu A, Kurtaran B, Komur S, Inal AS, et al *Rickettsia sibirica mongolitimonae* infection, Turkey 2016. Emerg Infect Dis. 2017;23(7):1214–6. doi: 10.3201/eid2307.170188 28628458 10.3201/eid2307.170188 PMC5512508

[17] Kuloglu F, Rolain JM, Akata F, Eroglu C, Celik AD, Parola P. Mediterranean Spotted Fever in the Trakya region of Turkey. Ticks Tick Borne Dis. 2012;3(5–6):298–304. doi: 10.1016/j.ttbdis.2012.10.030 23168048 10.1016/j.ttbdis.2012.10.030

[18] Gozalan A, Esen B, Rolain JM, Akin L, Raoult D. Is Q fever an emerging infection in Turkey? EMHJ. 2005;11(3):384–91. 16602458

[19] Saygi G. The epidemiology of toxoplasmosis in Turkey-a review of the literature. Wiad Parazytol. 2001:47, Suppl 1:19–30. 16897947

[20] Harman M. Cutaneous leishmaniasis. Turk J Dermatol. 2015;9:168–76. doi: 10.4274/tdd.2880

[21] Asman A, Solarz K, Cuber P, Casior T, Szilman P, Szilman E, et al Detection of protozoans *Babesia microti* and *Toxoplasma gondii* and their co-existence in ticks (Acari: Ixodida) collected in Tarnogorski district (Upper Silesia, Poland). Ann Agric Environ Med. 2015;22:80–3. doi: 10.5604/12321966.1141373 25780833 10.5604/12321966.1141373

[22] Trotta M, Nicetto M, Fogliazza A, Montarsi F, Caldin M, Furlanello T, et al Detection of *Leishmania infantum*, *Babesia canis* and *Rickettsiae* in ticks removed from dogs living in Italy. Ticks Tick Borne Dis. 2012;3(5–6):294–7. doi: 10.1016/j.ttbdis.2012.10.031 23182545 10.1016/j.ttbdis.2012.10.031

[23] Gureser AS, Akdogan O, Karadag F, Yapar D, Cebi K, Baykam N, et al. The epidemiologic features of patients with CCHF infection in an endemic region Corum, Turkey. 1st International Conference on Crimean-Congo Hemorrhagic Fever, Thessaloniki, Greece, 2015.

[24] Gargili A, Palomar AM, Midilli K, Portillo A, Kar S, Oteo JA. *Rickettsia* species in ticks removed from humans in Istanbul, Turkey. Vector-Borne Zoonotic Dis 2012;12[11]:938–41. doi: 10.1089/vbz.2012.0996 22925016 10.1089/vbz.2012.0996 PMC3491622

[25] Gulanber A, Gorenflot A, Schetters TPM, Carcy B. First molecular diagnosis of *Babesia vogeli* in domestic dogs from Turkey. Vet Parasitol. 2006;139(1–3):224–30. doi: 10.1016/j.vetpar.2006.02.035 16584843 10.1016/j.vetpar.2006.02.035

[26] Orkun O, Karaer Z, Cakmak A, Nalbantoglu S. Identification of tick-borne pathogens in ticks feeding on humans in Turkey. PLoS Negl Trop Dis. 2014;8(8):e3067 doi: 10.1371/journal.pntd.0003067 25101999 10.1371/journal.pntd.0003067 PMC4125308

[27] Bursali A, Keskin A, Keskin A, Kul-Koprulu T, Tekin S. Investigation on the presence of rickettsiae in ticks infesting humans in Corum. Turk Hij Den Biyol Derg. 2017;74(4):293–8. doi: 10.5505/TurkHijyen.2017.28291

[28] Filippova NA. Ixodid ticks of subfamily Amblyomminae. In: Fauna of Russia and neighboring countries Nauka Publishing House, St. Petersburg 1997 (in Russian).

[29] Walker JB, Keirans JE, Horak IG. The genus Rhipicephalus (Acari, Ixodidae): A guide to the brown ticks of the world Cambridge University Press, Cambridge 2000.

[30] Estrada-Pena A, Bouattour A, Camicas JL, Walker AR. Ticks of veterinary and medical importance: the Mediterranean basin. A Guide of Identification of Species University of Zaragoza Press, Zaragoza 2004.

[31] Kato CY, Chung IH, Robinson LK, Austin AL, Dasch GA, Massung RF. Assessment of Real-Time PCR assay for detection of *Rickettsia* spp. and *Rickettsia rickettsii* in banked clinical samples. J Clin Microbiol. 2013;51(1):314–7. doi: 10.1128/JCM.01723-12 23135935 10.1128/JCM.01723-12 PMC3536194

[32] Regnery RL, Spruill CL, Plikaytis BD. Genotypic identification of *Rickettsiae* and estimation of intraspecies sequence divergence for portions of two rickettsial genes. J Bacteriol. 1991;173(5):1576–89. 1671856 10.1128/jb.173.5.1576-1589.1991 PMC207306

[33] Bell CA, Patel R. A real-time combined polymerase chain reaction assay for the rapid detection and differentiation of *Anaplasma phagocytophilum*, *Ehrlichia chaffeensis* and *Ehrlichia ewingii*. Diagn Microbiol Infect Dis. 2005;53(4):301–6. doi: 10.1016/j.diagmicrobio.2005.06.019 16263231 10.1016/j.diagmicrobio.2005.06.019

[34] Jaton K, Peter O, Raoult D, Tissot JD, Greub G. Development of a high throughput PCR to detect *Coxiella burnetii* and its application in a diagnostic laboratory over a 7-year period. New Microbes New Infect. 2013;1(1):6–12. doi: 10.1002/2052-2975.8 25356317 10.1002/2052-2975.8 PMC4184484

[35] Diaz MH, Bai Y, Malania L, Winchell JM, Kosoy MY. Development of a novel genus-specific real-time PCR assay for detection and differentiation of *Bartonella* species and genotypes. J Clin Microbiol. 2012;50(5):1645–9. doi: 10.1128/JCM.06621-11 22378904 10.1128/JCM.06621-11 PMC3347110

[36] O’Rourke M, Traweger A, Lusa L, Stupica D, Maraspin V, Barrett PN, et al Quantitative detection of *Borrelia burgdorferi* sensu lato in erythema migrans skin lesions using internally controlled duplex real time PCR. PLOS one. 2013;16:8(5):e63968 doi: 10.1371/journal.pone.0063968 10.1371/journal.pone.0063968 PMC3655952 23696863

[37] Picken RN. Polymerase chain reaction primers and probes derived from flagellin gene sequences for specific detection of the agents of Lyme disease and North American relapsing fever. J Clin Microbiol. 1992;30:99–114. 1734073 10.1128/jcm.30.1.99-114.1992 PMC265004

[38] Versage JL, Severin DD, Chu MC, Petersen JM. Development of a multitarget real-time TaqMan PCR assay for inhanced detection of *Francisella tularensis* in complex specimens. J Clin Microbiol. 2003;41:5492–9. doi: 10.1128/JCM.41.12.5492-5499.2003 14662930 10.1128/JCM.41.12.5492-5499.2003 PMC309004

[39] Casati S, Sager H, Gern L, Piffaretti CJ. Presence of potentially pathogenic *Babesia sp*. for human in *Ixodes ricinus* in Switzerland. Ann Agric Environ Med. 2006;13:65–70. 16841874

[40] Toz SO, Culha G, Zeyrek FY, Ertabaklar H, Alkan MZ, Vardarli AT, et al A real-time ITS1-PCR based method in the diagnosis and species identification of Leishmania parasite from human and dog clinical samples in Turkey. PLoS Negl Trop Dis. 2013;7(5):e2205 doi: 10.1371/journal.pntd.0002205 23675543 10.1371/journal.pntd.0002205 PMC3649959

[41] Lin MH, Chen TC, Kuo TT, Tseng CC, Tseng CP. Real-Time PCR for quantitative detection of *Toxoplasma gondii*. J Clin Microbiol. 2000;38(11):4121–5. 11060078 10.1128/jcm.38.11.4121-4125.2000 PMC87551

[42] Kar S, Yilmazer N, Midilli K, Ergin S, Gargili A. *Borrelia burgdorferi* s.l. and *Rickettsia* spp. in ticks collected from European part of Turkey. Kafkas Univ Vet Fak Derg. 2013;19(1):19–24. doi: 10.9775/kvfd.2012.7033

[43] Kuloglu F, Rolain JM, Fournier PE, Akata F, Tugrul M, Raoult D. First isolation of *Rickettsia conorii* from humans in the Trakya (European) region of Turkey. Eur J Clin Microbiol Infect Dis. 2004;23(8):609–14. doi: 10.1007/s10096-004-1179-4 15278729 10.1007/s10096-004-1179-4

[44] Guner ES, Hashimoto N, Takada N, Kaneda K, Imai Y, Masuzawa T. First isolation and characterization of *Borrelia burgdorferi* sensu lato strains from *Ixodes ricinus* ticks in Turkey. J Med Microbiol. 2003;52(9):807–13. doi: 10.1099/jmm.0.05205–0 12909659 10.1099/jmm.0.05205-0

[45] Celebi B, Taylan Ozkan A, Kilic S, Akca A, Koenhemsi L, Pasa S, et al Seroprevalence of *Bartonella vinsonii* subsp. *berkhoffii* in urban and rural dogs in Turkey. J Vet Med Sci. 2010;72(11):1491–4. 20574140 10.1292/jvms.10-0188

[46] Arslan F, Karagoz E, Zemheri E, Vahaboglu H, Mert A. Tick-related facial cellulitis caused by *Francisella tularensis*. Infez Med. 2016;24(2):140–3. 27367325

[47] Sen E, Uchishima Y, Okamoto Y, Fukui T, Kadosaka T, Ohashi N et al Molecular detection of *Anaplasma phagocytophilum* and *Borrelia burgdorferi* in *Ixodes ricinus* ticks from Istanbul metropolitan area and rural Trakya (Thrace) region of north-western Turkey. Ticks Tick Borne Dis. 2011;2(2):94–8. doi: 10.1016/j.ttbdis.2011.03.004 21771542 10.1016/j.ttbdis.2011.03.004

[48] Aysul N, Karagenc T, Eren H, Aypak S, Bakirci S. Studies on tropical theileriosis in cattle from the province of Aydin and field evaluation of *Theileria annulata* schizonts. Türkiye Parazitol Derg. 2008;32(4):322–7. (in Turkish). 19156604

[49] Hosgor M, Bilgic HB, Bakirci S, Unlu AH, Karagenc T, Eren H. Detection of *Anaplasma/Ehrlichia* species of cattle and ticks in Aydin region. Türkiye Parazitol. Derg. 2015;39(4):291–8. doi: 10.5152/tpd.2015.4525 26809916 10.5152/tpd.2015.4525

[50] Gazi H, Ozkutuk N, Ecemis O, Atasoylu G, Koroglu G, Kurutepe S, Horasan GD. Seroprevalence of West Nile virus, Crimean-Congo hemorrhagic fever virus, *Francisella tularensis* and *Borrelia burgdorferi* in rural population of Manisa, western Turkey. J Vector Borne Dis. 2016;53(2):112–7. 27353580

[51] Aktas M, Ozubek S. Outbreak of anaplasmosis associated with novel genetic variants of *Anaplasma marginale* in a dairy cattle. Comp Immunol Microbiol Infect Dis. 2017;54:20–6. doi: 10.1016/j.cimid.2017.07.008 28915997 10.1016/j.cimid.2017.07.008

[52] Guclu HZ, Karaer KZ. Study on *Babesia caballi* (Nuttall, 1910) and *Theileria equi* in sporting and promotional horses living in the province of Ankara. Turkiye Parazitol Derg. 2007;31[2]:89–93 (in Turkish). 17594644

[53] Unver A, Rikihisa Y, Borku K, Ozkanlar Y, Hanedan B. Molecular detection and characterization of *Ehrlichia canis* from dogs in Turkey. Berl Munch Tierarztl Wochenschr. 2013;118(7–8):300–4. 16048040

[54] Celebi B, Carhan A, Kilic S, Babur C. Detection and genetic diversity of *Bartonella vinsonii* subsp. *berkhoffii* strains isolated from dogs in Ankara, Turkey. J Vet Med Sci. 2010;72(8):969–73. doi: 10.1292/jvms.09-0466 10.1292/jvms.09-0466 20234114

[55] Duzlu O, Inci A, Yildirim A, Onder Z, Ciloglu A. The investigation of vector-borne some protozoon and rickettsial infections in dogs by Real Time PCR and the molecular characterizations of the obtained isolates. Vet J Ankara Univ. 2014;61:275–82 (in Turkish).

[56] Keskin A, Bursali A, Keskin A, Tekin S. Molecular detection of spotted fever group *Rickettsiae* in ticks removed from humans in Turkey. Ticks Tick Borne Dis. 2016; 7(5):951–3. doi: 10.1016/j.ttbdis.2016.04.015 27131413 10.1016/j.ttbdis.2016.04.015

[57] Yesilyurt M, Kilic S, Cagasar O, Celebi B, Gul S. Two cases of tick-borne tularemia in Yozgat province, Turkey. Mikrobiyol Bul. 2011;45(4):746–54 (in Turkish). 22090307

[58] Orkun O, Emir H, Karaer Z. Ticks threatening lineage of Anatolian wild sheep (*Ovis gmelinii anatolica*) and determination of their tick-borne pathogens. Vet Parasitol 2016;228:77–84. doi: 10.1016/j.vetpar.2016.08.013 27692336 10.1016/j.vetpar.2016.08.013

[59] Guo H, Sevinc F, Ceylan O, Sevinc M, Ince E, Gao Y, et al A PCR survey of vector-borne pathogens in different dog populations from Turkey. Acta Parasitol. 2017;62(3):533–40. doi: 10.1515/ap-2017-0064 28682765 10.1515/ap-2017-0064

[60] Ekici OD, Sevinc F, Isik N. Instability of ovine babesiosis in an endemic area in Turkey. Vet Parasitol. 2012;188(3–4):372–5. doi: 10.1016/j.vetpar.2012.04.001 22538091 10.1016/j.vetpar.2012.04.001

[61] Kalkan K, Ozcelik S, Malatyali E. Seroprevalence of babesiosis in cattle from Sivas. doi: 10.1016/j.vetpar.2012.04.001 2010;34(1):11–6 (in Turkish). 20340080

[62] Tekin S, Dowd SE, Davinic M, Bursali A, Keskin A. Pyrosequencing based assessment of bacterial diversity in Turkish *Rhipicephalus annulatus* and *Dermacentor marginatus* ticks (Acari: Ixodidae). Parasitol Res. 2017;116(3):1055–61. doi: 10.1007/s00436-017-5387-0 28111714 10.1007/s00436-017-5387-0

[63] Aydin MF, Aktas M, Dumanli N. Molecular identification of *Theileria* and *Babesia* in ticks collected from sheep and goats in the Black Sea region of Turkey. Parasitol Res. 2015;114(1):65–9. doi: 10.1007/s00436-014-4160-x 25260692 10.1007/s00436-014-4160-x

[64] Poyraz O, Gunes T. Seroprevalance of *Babesia microti* in humans living in rural areas of the Sinop region. Turkiye Parazitol Derg. 2010;34(2):81–5. 20597050

[65] Altay K, Aydin MF, Dumanli N, Aktas M. Molecular detection of *Theileria* and *Babesia* infections in cattle. Vet Parasitol. 2008;158(4):295–301. doi: 10.1016/j.vetpar.2008.09.025 19008048 10.1016/j.vetpar.2008.09.025

[66] Aktas M, Ozubek S. Molecular and Parasitological survey of bovine piroplasms in the Black Sea region, including the first report of babesiosis associated with *Babesia divergens* in Turkey. J Med Entomol. 2015;52(6):1344–50. doi: 10.1093/jme/tjv126 26336265 10.1093/jme/tjv126

[67] Aktas M, Vatansever Z, Altay K, Aydin MF, Dumanli N. Molecular evidence for *Anaplasma phagocytophilum* in *Ixodes ricinus* from Turkey. Trans R Soc Trop Med Hyg. 2010;104 (1):10–5. doi: 10.1016/j.trstmh.2009.07.025 19744685 10.1016/j.trstmh.2009.07.025

[68] Aktas M, Altay K, Dumanli N. Molecular detection and identification of Anaplasma and *Ehrlichia* species in cattle from Turkey. Ticks Tick Borne Dis. 2011;2(1):62–5. doi: 10.1016/j.ttbdis.2010.11.002 21771539 10.1016/j.ttbdis.2010.11.002

[69] Aktas M, Altay K, Ozubek S, Dumanli N. A survey of ixodid ticks feeding on cattle and prevalence of tick-borne pathogens in the Black Sea region of Turkey. Vet Parasitol. 2012;187(3–4):567–71. doi: 10.1016/j.vetpar.2012.01.035 22365338 10.1016/j.vetpar.2012.01.035

[70] Cetinkol Y, Enginyurt O, Celebi B, Yildirim AA, Cankaya S, Aktepe OC. Investigation of zoonotic infections in risk groups in Ordu University Hospital, Turkey. Niger J Clin Pract. 2017;20(1):6–11. doi: 10.4103/1119-3077.181395 27958239 10.4103/1119-3077.181395

[71] Keskin A, Bursali A. Detection of *Rickettsia aeschlimannii* and *Rickettsia sibirica mongolitimonae* in *Hyalomma marginatum* (Acari: Ixodidae) ticks from Turkey. Acarologia. 2016;56(4):533–6. doi: 10.1051/acarologia/20164140

[72] Altay K, Aktas M, Dumanli N. Erzincan Yoresinde Sigirlarda *Theileria annulata* ve *Theileria buffeli*/*orientalis*’in Reverse Line Blotting Yontemi ile Arastirilmasi. Turkiye Parazitol Derg. 2007; 31(2):94–7. 17594645

[73] Oncel T, Vural G, Gicik Y, Arslan MO. Detection of *Babesia* (*Theileria*) *equi* (Laveran, 1901) in horses in the Kars province of Turkey. Turkiye Parazitol Derg. 2007;31(3):170–2. 17918051

[74] Sari B, Tasci GT, Kilic Y. Seroprevalence of *Dirofilaria immitis*, *Ehrlichia canis* and *Borrelia burgdorferi* in dogs in Igdir Province, Turkey. Kafkas Univ Vet Fak Derg. 2013;19(5):735–9, doi: 10.9775/kvfd.2012.8466

[75] Kilic A, Kalender H, Koc O, Kilinc Irehan B, Berri M. Molecular investigation of *Coxiella burnetii* infections in aborted sheep in eastern Turkey. Iran J Vet Res. 2016;17(1):41–4. 27656228 PMC4898019

[76] Aktas M, Altay K, Dumanli N, Kalkan A. Molecular detection and identification of *Ehrlichia* and *Anaplasma* species in ixodid ticks. Parasitol Res. 2009;104(5):1243–8. doi: 10.1007/s00436-009-1377-1 19247690 10.1007/s00436-009-1377-1

[77] Cikman A, Aydin M, Gulhan B, Karakecili F, Ozcicek A, Kesik OA, et al The seroprevalence of *Coxiella burnetii* in Erzincan, Turkey: Identification of the risk factors and their relationship with geographical features. J Vector Borne Dis. 2017;54(2):157–63. 28748837

[78] Guven E, Avcioglu H, Cengiz S, Hayirli A. Vector-borne pathogens in stray dogs in Northeastern Turkey. Vector-Borne Zoonotic Dis. 2017; 17(8):610–7. doi: 10.1089/vbz.2017.2128 28632488 10.1089/vbz.2017.2128

[79] Altay K, Aktas M, Dumanli N. Detection of *Babesia ovis* by PCR in *Rhipicephalus bursa* collected from naturally infested sheep and goats. Res Vet Sci. 2008;85(1):116–9. doi: 10.1016/j.rvsc.2007.08.002 17881019 10.1016/j.rvsc.2007.08.002

[80] Guven E, Avcioglu H, Deniz A, Balkaya I, Abay U, Yavuz S, et al Prevalence and molecular characterization of *Theileria equi* and *Babesia caballi* in jereed horses in Erzurum, Turkey. Acta Parasitol. 2017;62(1):207–13. doi: 10.1515/ap-2017-0025 28030350 10.1515/ap-2017-0025

[81] Ozubek S, Aktas M. Molecular and parasitological survey of ovine piroplasmosis, including the first report of *Theileria annulata* (Apicomplexa: Theileridae) in sheep and goats from Turkey. J Med Entomol. 2017;54(1):212–20. doi: 10.1093/jme/tjw134 28082649 10.1093/jme/tjw134

[82] Aktas M, Ozubek S. A survey of canine haemoprotozoan parasites from Turkey, including molecular evidence of an unnamed *Babesia*. Comp Immunol Microbiol Infect Dis. 2017; 52:36–42. doi: 10.1016/j.cimid.2017.05.007 28673460 10.1016/j.cimid.2017.05.007

[83] Aktas M, Ozubek S, Ipek DN. Molecular investigation of *Hepatozoon* species in dogs and developmental stages in *Rhipicephalus sanguineus*. Parasitol Res. 2013; 112(6):2381–5. doi: 10.1007/s00436-013-3403-6 23535887 10.1007/s00436-013-3403-6

[84] Keskin A, Keskin A, Bursali A, Tekin S. Ticks (Acari: Ixodida) parasitizing humans in Corum and Yozgat provinces, Turkey. Exp Appl Acarol. 2015;67(4):607–16. doi: 10.1007/s10493-015-9966-4 26385209 10.1007/s10493-015-9966-4

[85] Keskin A, Bulut YE, Keskin A, Bursali A. Tick attachment sites in humans living in the Tokat province of Turkey. Turk Hij Den Biyol Derg. 2017;74(2): 121–8. doi: 10.5505/TurkHijyen.2017.24993

[86] Rizzoli A, Silaghi C, Obiegala A, Rudolf I, Hubálek Z, Foldvári G, et al *Ixodes ricinus* and its transmitted pathogens in urban and peri-urban areas in Europe: New hazards and relevance for public health. Front Public Health. 2014;2:251 doi: 10.3389/fpubh.2014.00251 25520947 10.3389/fpubh.2014.00251 PMC4248671

[87] Keskin A. A systematic study on the ticks (Acari: Ixodida) of Corum and Yozgat provinces and determination of the presence of the Rickettsia in these ticks by using PCR. Master Thesis, Gaziosmanpasa University, Graduate School of Natural and Applied Sciences, 2015.

[88] Orkun O, Karaer Z, Cakmak A, Nalbantoglu S. Spotted fever group *Rickettsiae* in ticks in Turkey. Ticks Tick Borne Dis. 2014;5(2):213–8. doi: 10.1016/j.ttbdis.2012.11.018 24355764 10.1016/j.ttbdis.2012.11.018

[89] Raoult D, Fournier PE, Abboud P, Caron F. First documented human *Rickettsia aeschlimannii* infection. Emerg Infect Dis. 2002;8:748–9. doi: 10.3201/eid0807.010480 12095451 10.3201/eid0807.010480 PMC2730330

[90] Parola P, Rovery C, Rolain JM, Brouqui P, Davoust B, Raoult D. *Rickettsia slovaca* and *R*. *raoultii* in tick-borne rickettsioses. Emerg Infect Dis. 2009;15(7):1105–8. doi: 10.3201/eid1507.081449 19624931 10.3201/eid1507.081449 PMC2744242

[91] Parola P, Paddock CD, Socolovschi C, Labruna MB, Mediannikov O, Kernif T, et al Update on tick-borne rickettsioses around the world: A geographic approach. Clin Microbiol Rev. 2013;26(4):657–702. doi: 10.1128/CMR.00032-13 24092850 10.1128/CMR.00032-13 PMC3811236

[92] Kleinerman G, Baneth G, Mumcuoglu KY, van Straten M, Berlin D, Apanaskevich DA, et al Molecular detection of *Rickettsia africae*, *Rickettsia aeschlimannii* and *Rickettsia sibirica* mongolitimonae in camels and *Hyalomma* spp. ticks from Israel. Vector-Borne Zoonotic Dis. 2013;13(12):851–6. doi: 10.1089/vbz.2013.1330 24107206 10.1089/vbz.2013.1330

[93] de Sousa R, Barata C, Vitorino L, Santos-Silva M, Carrapato C, Torgal J, et al *Rickettsia sibirica* isolation from a patient and detection in ticks, Portugal. Emerging Infect Dis. 2006;12:1103–8. doi: 10.3201/eid1207.051494 16836827 10.3201/eid1207.051494 PMC3291052

[94] Rar V, Golovljova I. *Anaplasma*, *Ehrlichia*, and "*Candidatus* Neoehrlichia" bacteria: pathogenicity, biodiversity, and molecular genetic characteristics, A review. Infect Genet Evol. 2011;11(8):1842–61. doi: 10.1016/j.meegid.2011.09.019 21983560 10.1016/j.meegid.2011.09.019

[95] Parola P, Raoult D. Ticks and tickborne bacterial diseases in humans: an emerging infectious threat. Clin Inf Dis. 2001;32:897–928. doi: 10.1086/319347 11247714 10.1086/319347

[96] Sreter-Lancz Szell Z, Sreter T, Marialigeti K. Detection of novel *Francisella* in *Dermacentor reticulatus*: A need for careful evaluation of PCR-based identification of *Francisella tularensis* in Eurasian ticks. Vector Borne Zoonotic Dis. 2009;9(1):123–5. doi: 10.1089/vbz.2008.0010 18945184 10.1089/vbz.2008.0010

[97] Gurcan S. Epidemiology of tularemia. Balkan Med J. 2014;31(1):3–10. doi: 10.5152/balkanmedj.2014.13117 25207161 10.5152/balkanmedj.2014.13117 PMC4115998

[98] Duzlu O, Yildirim A, Inci A, Gumussoy KS, Ciloglu A, Onder Z. Molecular investigation of *Francisella*-like endosymbiont in ticks and *Francisella tularensis* in ixodid ticks and mosquitoes in Turkey. Vector Borne Zoonotic Dis. 2016;16(1):26–32. doi: 10.1089/vbz.2015.1818 26741324 10.1089/vbz.2015.1818

[99] Angelakis E, Pulcini C, Waton J, Imbert P, Socolovschi C, Edouard S, et al Scalp eschar and neck lymphadenopathy caused by *Bartonella henselae* after tick bite. Clin Infect Dis. 2010;50(4):549–51. doi: 10.1086/650172 20070235 10.1086/650172

[100] Hildebrandt A, Gray JS, Hunfeld KP. Human babesiosis in Europe: What clinicians need to know. Infection. 2013;41:1057–72. doi: 10.1007/s15010-013-0526-8 24104943 10.1007/s15010-013-0526-8

[101] Adelson ME, Rao RV, Tilton RC, Cabets K, Eskow E, Fein L, et al Prevalence of *Borrelia burgdorferi*, *Bartonella* spp., *Babesia microti*, and *Anaplasma phagocytophila* in *Ixodes scapularis* ticks collected in Northern New Jersey. J Clin Microbiol. 2004;42(6):2799–2801. doi: 10.1128/JCM.42.6.2799-2801.2004 10.1128/JCM.42.6.2799-2801.2004 PMC427842 15184475

[102] Wójcik-Fatla A, Zając V, Sawczyn A, Cisak E, Dutkiewicz J. *Babesia* spp. in questing ticks from eastern Poland: Prevalence and species diversity. Parasitol Res. 2015;114(8):3111–6. doi: 10.1007/s00436-015-4529-5 25976982 10.1007/s00436-015-4529-5 PMC4513193

[103] Aktas M, Vatansever Z, Ozubek S. Molecular evidence for trans-stadial and transovarial transmission of *Babesia occultans* in *Hyalomma marginatum* and *Rhipicephalus turanicus* in Turkey. Vet Parasitol. 2014;29;204(3–4):369–71. doi: 10.1016/j.vetpar.2014.05.037 25002306 10.1016/j.vetpar.2014.05.037

[104] Tuzdil AN. First *Hepatozoon canis* case in the country. Turk Baytarlar Cemiyeti Mecmuasi. 1933;13:35. (in Turkish).

[105] Robinson PM. Theileriosis annulata and its transmission-a review. Trop Anim Health Prod. 1982;14(1):3–12. 6805112 10.1007/BF02281092

[106] Siroký P, Kamler M, Modrý D. Long-term occurrence of *Hemolivia* cf. *mauritanica* (Apicomplexa: Adeleina: Haemogregarinidae) in captive *Testudo marginata* (Reptilia: Testudinidae): evidence for cyclic merogony? J Parasitol. 2004;90(6):1391–3. doi: 10.1645/GE-3306 15715234 10.1645/GE-3306

