# Supplementary figures and images for: Bacterial and protozoal pathogens found in ticks collected from humans in Corum province of Turkey
Source: PLoS Negl Trop Dis. 2018 Apr 12;12(4):e0006395. doi: 10.1371/journal.pntd.0006395 (PMC5916866; doi:10.1371/journal.pntd.0006395)

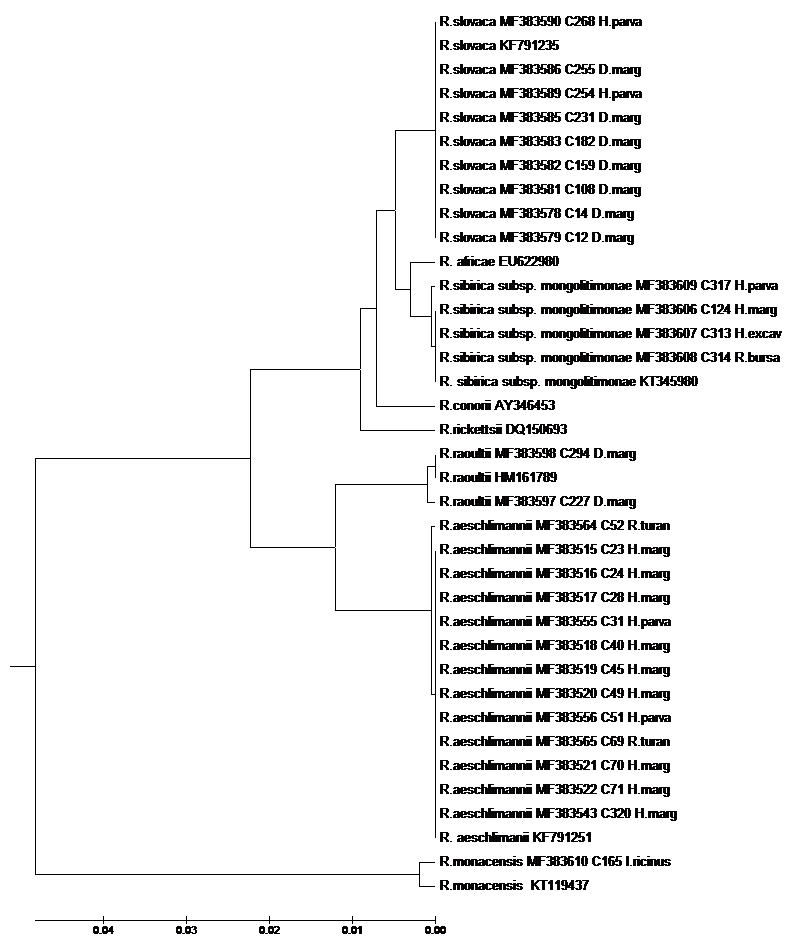

Supplement: S1 Fig — Phylogenetic tree based on aligned sequences of the rickettsial ompA gene, constructed using UPMGA in MEGA5.1 software. GenBank accession numbers of the Rickettsiae are given after the names of bacteria. (TIF) [file pntd.0006395.s001.tif]

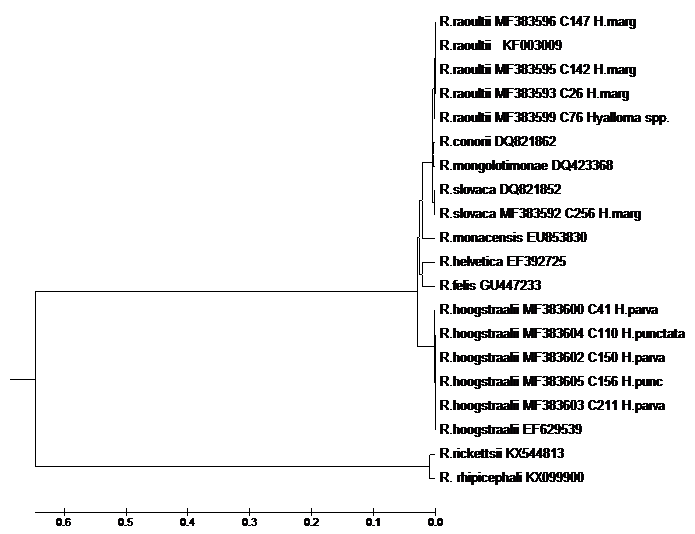

Supplement: S2 Fig — Phylogenetic tree based on aligned sequences of the rickettsial gltA gene, constructed using UPGMA in MEGA5.1 software. GenBank accession numbers of sequences are given after the names of bacteria. (TIF) [file pntd.0006395.s002.tif]

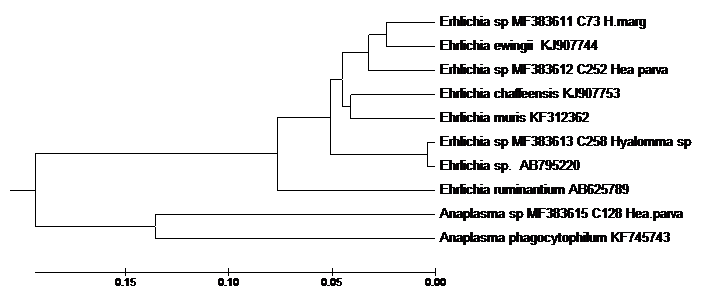

Supplement: S3 Fig — Phylogenetic tree based on aligned sequences of the heat shock protein (groEL) gene, constructed using UPGMA in MEGA5.1 software. GenBank accession numbers of sequences are given after the names of bacteria. (TIF) [file pntd.0006395.s003.tif]

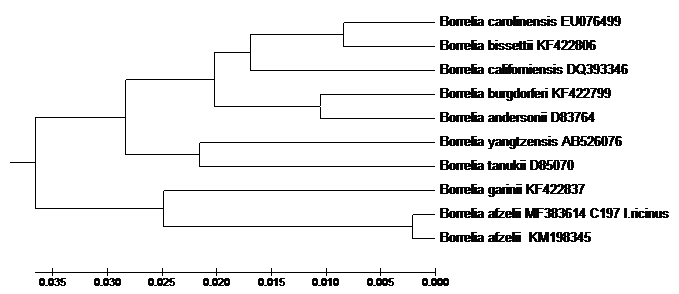

Supplement: S4 Fig — Phylogenetic tree based on aligned sequences of the Borrelia flaB gene, constructed using UPGMA in MEGA5.1 software. GenBank accession numbers of sequences are given after the names of bacteria. (TIF) [file pntd.0006395.s004.tif]

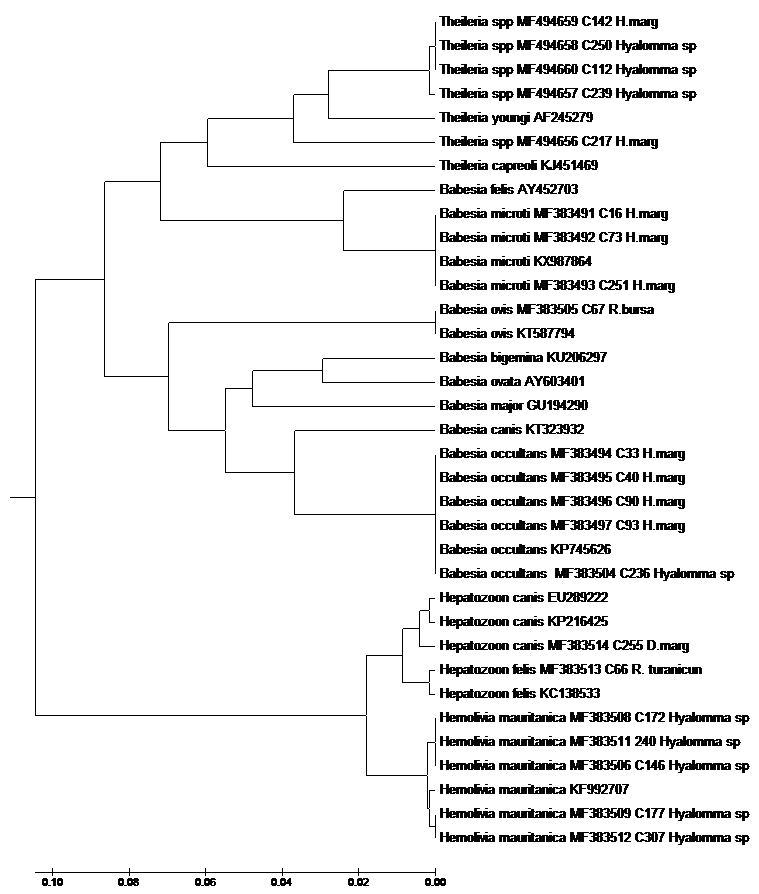

Supplement: S5 Fig — Phylogenetic tree based on aligned sequences of 18S ribosomal RNA gene, constructed using UPGMA in MEGA5.1 software. GenBank accession numbers of sequences are given after the names of the protozoa. (TIF) [file pntd.0006395.s005.tif]
